# Three-dimensional visualization of multiple synapses in thick sections using high-voltage electron microscopy in the rat spinal cord

**DOI:** 10.1016/j.dib.2015.07.005

**Published:** 2015-07-26

**Authors:** Keita Satoh, Keiko Takanami, Kazuyoshi Murata, Mitsuhiro Kawata, Tatsuya Sakamoto, Hirotaka Sakamoto

**Affiliations:** a Ushimado Marine Institute, Graduate School of Natural Science and Technology, Okayama University, Ushimado, Setouchi, Okayama 701-4303, Japan; b Department of Anatomy and Neurobiology, Kyoto Prefectural University of Medicine, Kawaramachi-Hirokoji, Kamigyo-ku, Kyoto 602-8566, Japan; c National Institute for Physiological Sciences, Nishigonaka, Myodaiji, Okazaki 444-8585, Japan

## Abstract

This data article contains complementary figure and movies (Supplementary Movies 1–3) related to the research article entitled, “Effective *synaptome* analysis of itch-mediating neurons in the spinal cord: a novel immunohistochemical methodology using high-voltage electron microscopy” [7]. It is important to show the synaptic connections at the ultrastructural level to understand the neural circuit, which requires the three-dimensional (3-D) analyses in the electron microscopy. Here, we applied a new sample preparation method, a high-contrast en bloc staining according to the protocol of the National Center for Microscopy and Imaging Research (NCMIR), University of California, San Diego, CA, USA to high-voltage electron microscopy (HVEM) tomography in order to examine the 3-D chemical neuroanatomy of the rat spinal cord. Pre-embedding immunoelectron microscopy was used in this study. HVEM has an excellent potential to directly visualize the ultrastructures in semi-thin sections (~5 μm thick), and we have successfully visualized many itch-mediating synaptic connections and neural networks in the spinal cord using “HVEM tomography”. Moreover, the methodology used in this study is simple and can be applied in multiple ways. This is an important contribution to ultrastructural investigations of the central nervous system in the present post-genomic age.

## Specifications table

**Table:** 

Subject area	*Biology*
More specific subject area	*Neuroscience*
Type of data	*Images and movies*
How data was acquired	*Using high-voltage electron microscopy (HVEM) (Hitachi H-1250M at the National Institute for Physiological Sciences, Okazaki, Japan). Computer tomography analyses were performed with Chimera (University of California, San Francisco; UCSF, CA, USA).*
Data format	*Images were processed with IMOD, UCSF Chimera and the AMIRA software packages.*
Experimental factors	*Three-dimensional (3-D) analyses were performed. Immunostained semi-thin sections were observed with HVEM by tilting the specimen from −60.0° to +60.0°, continuously. These 121 serial-tilted images were digitized and 3-D models were constructed using IMOD software. Animations and ultrastructural analyses were performed with UCSF Chimera software and manually segmented using the AMIRA software package.*
Experimental features	*We applied a high-contrast en bloc staining to HVEM tomography.*
Data source location	*Not applicable*
Data accessibility	*The data are supplied with this article*

## Value of the data

• Application of NCMIR method en bloc to high-voltage electron microscopy (HVEM) tomography allows a fine membranous visualization of the three-dimensional (3-D) structure in semi-thin sections at the ultrastructural level.
• This new methodology using HVEM tomography combined with immunohistochemistry is useful for the 3-D analysis of both synaptic connections and chemical neuroanatomy at the ultrastructural level, and this is potentially a new research approach/alternative for many laboratories.
• Using HVEM tomography, we demonstrated a previously uncharacterized itch-mediating neural network.
• The HVEM methodology can be widely and easily applied in multiple ways at either the cellular and organotypic levels.

## 1 Data, experimental design, materials and methods

### 1.1 Animals

Adult male Wistar rats were obtained from the Charles River Laboratories Japan (Yokohama, Japan). All experimental procedures have been authorized by the Committees for Animal Research, Okayama University and Kyoto Prefectural University of Medicine, Japan.

### 1.2 Immunohistochemistry for high-voltage electron microscopy (HVEM)

**Fig. 1:**
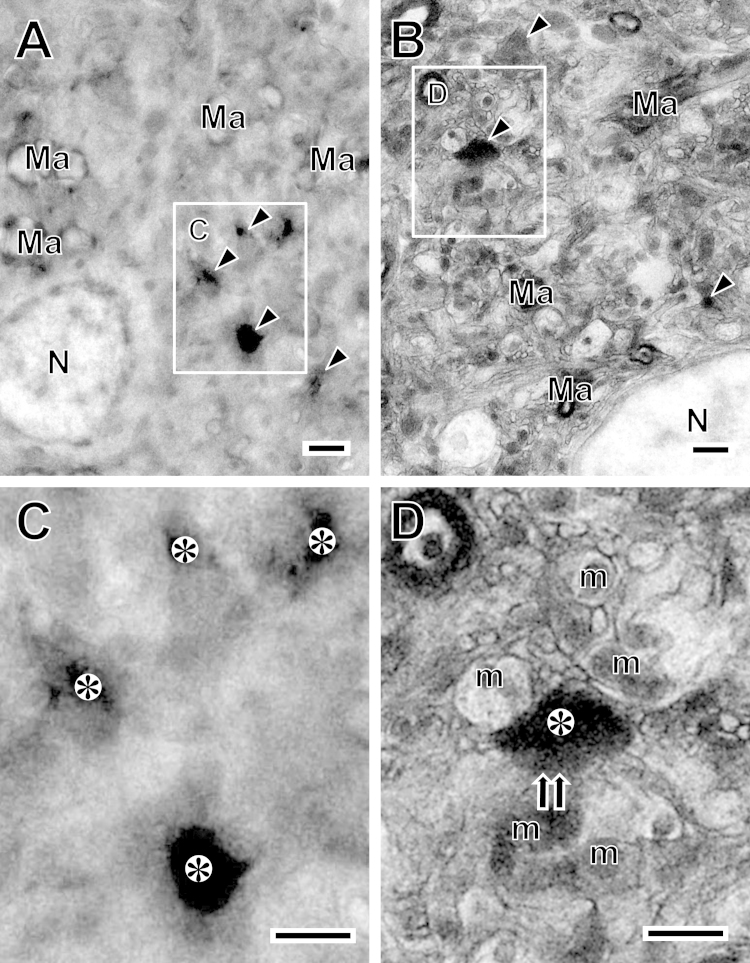
HVEM images are obtained from semi-thin sections, which are contrasted with the heavy metal staining (A) or the NCMIR method (B). Arrowheads in (A) and (B) indicate possible GRP-positive terminals. The boxed areas in (A) and (B) are enlarged in (C) and (D), respectively. (C) The enlarged area shows that GRP-positive terminals (asterisks) are contrasted, but other membranous structures are unclear. (D) In contrast, the fine membranous structures include the electron-dense GRP-positive presynaptic terminal (asterisk), synaptic connection (double arrows), and surrounding structures without any additional heavy metal staining after the NCMIR method en bloc. Ma: myelinated axon; m: mitochondrion; N: nucleus. Scale bars=1 μm.

Male rats (*n*=3) were overdosed with sodium pentobarbital and perfusion fixed with 4% paraformaldehyde, 0.1% glutaraldehyde, and 0.3% tannic acid in 0.1 M phosphate buffer. Cervical spinal cords were immediately removed and immersed in 4% paraformaldehyde in 0.1 M phosphate buffer for 3 h. Spinal sections (C3–C6 level; 30 µm in thickness) were prepared with a LinearSlicer^®^ (Dosaka EM, Kyoto, Japan). Immunohistochemistry for gastrin-releasing peptide (GRP) was performed in this study according to our previous method [5,6,8]. The free-floating sections were thoroughly washed with phosphate buffered saline (PBS) and preincubated in PBS containing 0.05% Triton X-100, 1% normal goat serum, and 1% BSA for 30 min at room temperature to block nonspecific reactions. Sections were then incubated with the primary rabbit antiserum against rat GRP_20–29_ (1:1000) (AssayPro, St. Charles, MO, USA) overnight at room temperature with gentle agitation. The GRP antiserum used in this study have previously shown to be specific for rat GRP in the spinal cord [7,8]. Immunoreactive (ir) products were detected with a streptavidin–biotin kit (Nichirei, Tokyo, Japan), followed by diaminobenzidine development, as described previously [7,8]. The sections were dehydrated and flat embedded in epoxy resin (Quetol-812; Nisshin EM, Tokyo, Japan) as previously described [3,5,8]. Subsequently, semi-thin sections (1–2 µm in thickness) containing the GRP-positive fibers in the dorsal horn (DH) of the cervical spinal cord were then prepared and collected on mesh grids coated with a collodion film. Each grid-mounted semi-thin section was first selected using a light microscope (Olympus; BH-2, Tokyo, Japan). Selected grids or sections were observed using an HVEM (Hitachi H-1250M; National Institute for Physiological Sciences, Okazaki, Japan) at an accelerating voltage of 1000 kV (Fig. 1).

### 1.3 En bloc staining (NCMIR method) for HVEM tomography

The GRP-immunostained sections were then subjected to special en bloc staining according to the protocol of the National Center for Microscopy and Imaging Research (NCMIR), University of California, San Diego, CA, USA with slight modifications [1]. The NCMIR method is widely used for serial block-face SEM (SBF-SEM), which was designed to enhance signal for backscatter electron imaging of epoxy-embedded mammalian tissue at low accelerating voltages [1]. This sample preparation method for effective heavy metal staining was applied to our *synaptome* analysis by HVEM tomography. Briefly, after being washed with 0.15 M cacodylate buffer, the sections were post-fixed for 1 h in 2% aqueous osmium tetroxide/1.5% potassium ferrocyanide in 0.15 M cacodylate buffer, for 20 min in a thiocarbohydrazide solution, and then for 30 min in a 2% osmium tetroxide solution. Sections were placed in 1% uranyl acetate overnight at 4 °C and then in a lead aspartate solution in a 60 °C oven for 30 min.

### 1.4 Tomography

The specimen was tilted from −60.0° to +60.0° and imaged at 1° steps (121 images per view; 0.0, ±1.0 ~±60.0). The images were digitized and 3-D models were constructed using IMOD software [2,4]. Individual subcellular and organelle structures were observed by UCSF Chimera and manually segmented using the AMIRA software package (FEI Visualization Science Group, Burlington, MA, USA) [4]. This software package was also used to generate the 3-D image figures (Fig. 1).

## 2 Conflict of interests

The authors declare that there are no potential conflicts of interest.

## Appendix A Supplementary materials

Supplementary Movie 1. Representative movie prepared by tomography analysis using high-voltage electron microscopy. A total of 121 images were obtained by tilting the specimen from −60.0° to +60.0°, continuously. The 3-dimensional reconstruction was performed from these 121 serial-tilted images. The tilting axis is vertical.

Supplementary Movie 2. High-voltage electron microscopy tomography analysis showed that gastrin-releasing peptide-positive presynaptic terminals (green) formed a varicose-like structure, and each varicose-like structure tended to form a synapse (red) with dendrite-like structure of postsynaptic neurons (blue).

Supplementary Movie 3. Gastrin-releasing peptide-positive presynaptic terminals (green) connected with postsynaptic neurons (blue) with postsynaptic density (red). These terminals (green) formed a varicose-like structure, and each varicose-like structure tended to contain some mitochondria (magenta).

## References

[1] T. Deerinck, E. Bushong, A. Thor, M. Ellisman, NCMIR methods for 3D EM: a new protocol for preparation of biological specimens for serial block face scanning electron microscopy, 2010, 〈http://ncmir.ucsd.edu/sbfsem-protocol.pdf〉

[2] Kremer J.R., Mastronarde D.N., McIntosh J.R. (1996). Computer visualization of three-dimensional image data using IMOD. J. Struct. Biol..

[3] Oti T., Satoh K., Saito K., Murata K., Kawata M., Sakamoto T., Sakamoto H. (2012). Three-dimensional evaluation of the spinal local neural network revealed by the high-voltage electron microscopy: a double immunohistochemical study. Histochem. Cell Biol..

[4] Pettersen E.F., Goddard T.D., Huang C.C., Couch G.S., Greenblatt D.M., Meng E.C., Ferrin T.E. (2004). UCSF Chimera—a visualization system for exploratory research and analysis. J. Comput. Chem..

[5] Sakamoto H., Arii T., Kawata M. (2010). High-voltage electron microscopy reveals direct synaptic inputs from a spinal gastrin-releasing peptide system to neurons of the spinal nucleus of bulbocavernosus. Endocrinology.

[6] Sakamoto H., Matsuda K.-I., Zuloaga D.G., Hongu H., Wada E., Wada K., Jordan C.L., Breedlove S.M., Kawata M. (2008). Sexually dimorphic gastrin releasing peptide system in the spinal cord controls male reproductive functions. Nat. Neurosci..

[7] Satoh K., Takanami K., Murata K., Kawata M., Sakamoto T., Sakamoto H. (2015). Effective synaptome analysis of itch-mediating neurons in the spinal cord: a novel immunohistochemical methodology using high-voltage electron microscopy. Neurosci. Lett..

[8] Takanami K., Sakamoto H., Matsuda K.I., Satoh K., Tanida T., Yamada S., Inoue K., Oti T., Sakamoto T., Kawata M. (2014). Distribution of gastrin-releasing peptide in the rat trigeminal and spinal somatosensory systems. J. Comp. Neurol..

